# Effect of a waste management intervention program on knowledge, attitude, and practice (KAP) of nurses and housekeepers: a quasi-experimental study, Egypt

**DOI:** 10.1186/s42506-023-00140-w

**Published:** 2023-08-04

**Authors:** Eman Khashaba, Abdel Hady El-Gilany, Khadija Denewar

**Affiliations:** Public Health and Community Medicine Department, Faculty of Medicine, Mansoura University, Mansoura, Egypt

**Keywords:** Waste management, Intervention, Training-healthcare workers, Healthcare waste

## Abstract

**Background:**

Improper healthcare waste management practice is alarming in developing countries because resources are inadequate and waste management is often delegated to poorly educated and untrained laborers. This study aimed to compare the pre-KAP versus post-KAP towards the waste management program for nurses and housekeepers. In addition, it aimed to explore possible factors affecting the pre- and post-KAP in Mansoura Emergency University Hospital, Egypt.

**Subjects and methods:**

One hundred thirty-three newly employed nurses, housekeepers, and those who need refreshment training as nominated by head nurses and link occupational health and safety nurses in the hospital were recruited for the study. The study’s intervention included multiple training sessions using a PowerPoint presentation in Arabic with appropriate illustrations followed by an open discussion. An Arabic self-administered questionnaire containing demographic and occupational history, knowledge (27 questions), attitude (10 questions), and practice (9 questions) was used pre- and post-intervention.

**Results:**

The overall KAP scores among the studied healthcare workers were significantly higher after the intervention. The pre- and post-knowledge scores were significantly different with respect to education, job description, and duration of employment (*p* < 0.05). The post-attitude scores were significantly different with respect to education and job description only (*p* < 0.05). The total pre-practice scores were significantly different with respect to education and job description (*p* < 0.05). However, the post-practice scores were significantly different with respect to sex, age, education, and job description (*p* < 0.05).

**Conclusion:**

There was a significant improvement in the KAP scores post-intervention. The post-knowledge and attitude scores were significantly better in nurses and participants with a higher education. The post-practice score was significantly better for females, participants with an age ≥ 30 years, higher education, and nursing jobs. The combination of training and supervision was crucial for the success of waste management programs. Higher education levels are required for housekeepers to be capable of gaining better knowledge, follow rules, and be ready for any challenges in the future.

## Introduction

Healthcare facilities such as hospitals, clinics, blood banks, and laboratories generate medical waste. Human or animal tissue, blood or other bodily fluids, excretions, medications or pharmaceutical items, swabs or dressings, syringes, and needles or other sharp tools may all be included. These represent biological, chemical, and multiple safety hazards to any person coming in contact with them [1].

Poor healthcare waste management (HCWM) practices may result in patients, staff, waste handlers, and the community being exposed to the unnecessary health risks of the waste [2]. Improper HCWM practice is alarming in developing countries because resources are inadequate to manage waste, and waste management is often delegated to poorly educated and untrained workers, who perform without proper guidance or adequate protection [3, 4].

Egypt, like many developing countries, is struggling to improve its hospital waste management standards. Despite the fact that the Environmental Law No. 4 of 1994 and lately the Waste Management Regulation Law No. 202 of 2020 and its executive regulations were enacted to organize the implementation of integrated hospital waste management, both healthcare staff and authorities are unable to create efficient mechanisms for the segregation, collection, transport, or treatment due to inadequate legislative compliance and enforcement. Because of mal-distribution and missing awareness of HCWM guidelines, healthcare personnel in Egypt are frequently uninformed of this regulation. The most common means of hazardous healthcare waste treatment are incineration or autoclaving. In Egypt, the majority of general hospitals utilize incineration, while the majority of teaching and university hospitals use autoclaving [5]. Healthcare workers (HCWs) play an important role in bio-medical waste management due to their expertise, attitude, and practices (KAP) [6].

The waste management program is an ongoing training since 2015 as a part of continuous training courses in the Mansoura University Emergency Hospital that is provided twice a year to nurses, housekeepers, and laboratory technicians. However, there are no previous studies assessing the effects of these training on KAP of the target population.

The aim of this study is to compare the pre- with the post-intervention knowledge, attitude, and practice (KAP) of nurses and housekeepers towards waste management and factors associated with its variation.

## Methods

### Study design and setting

This quasi-experimental study was done in Mansoura University Emergency Hospital from November 2021 to March 2022. This is the only emergency hospital in the Delta region specialized in trauma and accidents and recently triage and isolation of COVID-19 patients. There is a high rate of flow of patients and also a considerable turnover rate for nurses and housekeepers due to excessive workload.

### Study subjects

All newly employed nurses and housekeepers as well as those who need refreshment training as nominated by head nurses were recruited in the study. These job categories participate in healthcare waste management under the supervision of the waste management committee. Nurses are responsible for waste segregation in patients’ wards, the emergency department, operative rooms, and intensive care units. Housekeepers are responsible for the collection, transportation, and storage of infectious and non-infectious waste in temporary storage rooms.

### Sample size

All target HCWs attended the training course (*n* = 165) on different sequential sessions (twice per month). Each session included approximately 30–35 HCWs. One hundred thirty three out of 165 (80.6%) attended the training program and completed both pre- and post-KAP questionnaires. Non-responders were excluded due to incomplete post-KAP data.

### Study tool

An Arabic self-administered questionnaire containing demographic data, occupational history, previous history of HCV infection, history of Hepatitis B vaccination, previous training about waste management and knowledge (27 questions), attitude (10 questions), and practice (9 questions) was used. The questionnaire was developed by the researchers after an extensive literature review including the Egyptian manual guide for healthcare facility waste management produced by the Ministry of Environmental Affairs (2015) [7].

The questionnaire was completed twice: before and 2 weeks after the intervention program, to check the changes of knowledge, attitude and practice. The score of each item was calculated according to a specific coding system (correct answer = 1, wrong answer = 0).

***Description:*** Knowledge score (27 questions) (mix between yes/no predefined answers and multiple choices) had a total score ranging from 0 to 27. The questions included concepts and definitions in waste management such as medical waste definition, hazardous waste, waste management, health effects of hazardous waste, different types of healthcare waste, way of segregation of this waste, collection of different types of waste, requirements for appropriate storage rooms, and knowledge of dealing with improper segregation.

Attitude score (10 statements) (yes, no, do not know) had a total score ranging from 0 to 10. The score included waste segregation, sharp objects and chemical material collection, requirement for the storage room and transportation containers, attending training programs, and visual screening of red bags.

Practice score (9 questions on Likert scale (always-most of times-sometimes-never) had a total score ranging from 0 to 9. The practice score included questions about performing waste segregation, actively participating in waste management training programs, following rules of administration with regards to the collection of hazardous waste, reporting of needle stick injuries, recapping needles before collection, getting rid of needles in red bags, removing the infectious waste from black bags, wearing the personal protective equipment during disinfection, and cleaning and removal of biological fluid spills. The time required to complete the questionnaire ranges from 15 to 20 min.

### Study intervention

The intervention program was prepared by the authors. The intervention included multiple training sessions (5 sessions) using a PowerPoint presentation in Arabic with appropriate illustration pictures (1.5 h each) followed by an open discussion. The lecture content was uploaded to the hospital website and made available to the occupational health and safety team in the hospital. The training of workers handling waste was presented in Arabic in the form of simple instructions and photos to impart the correct knowledge and practice with respect to waste management, as well as the correction of faulty practices. This was presented after an introduction highlighting the definition and types of hazardous waste, classification of healthcare waste, impact of waste on environment, principles of waste segregation, collection, transportation, storage, and treatment before final disposal. In addition, occupational health and safety practice with regard to the proper response to needle stick/sharp objects injuries and blood and/or biological fluids spills, biological hazards, protective hepatitis B virus vaccinations, and finally requirements for transportation containers and temporary storage rooms [7].

Both the questionnaire and the intervention program were subjected to a jury of five experts in waste management and occupational health to test the contents and validity of the KAP tools. The comments of experts were taken into consideration and a consensus was obtained. The content validity indices of the KAP tools ranged from 0.7 to 1 per item for their relevance and clarity and from 0.6 to 100 per expert. Reliability of the study tools was tested in a pilot study on 30 lab technicians not included in the full-scale study. The Cronbach’s alphas of internal consistency were 0.646, 0.737, and 0.769 for the knowledge, attitude, and practice scales respectively.

### Statistical analysis

Data were analyzed using the statistical package of the social science statistical program software version 22 (SPSS Inc., Chicago, USA). Quantitative variables were summarized using mean or median according to the data distribution. Qualitative variables were summarized with number and percentage. Paired *t* test was used for pre- and post-comparisons. The Independent *t* test was used to compare quantitative variables in two different groups. One-way ANOVA was used to compare quantitative variables; as appropriate. The level of statistical significance was set to 0.05.

## Results

### Characteristics of study participants

The mean age of studied subjects was 38.4 (± 9.5) years and most of them were females (81.2%) and only 60.3% had received the Hepatitis B vaccine. A little less than two thirds (63.9%) had technical education. Less than half of them (45.9%) had received previous waste management training (Table 1).

**Table 1:** Demographic characteristics of waste management workers participating in the training intervention, Mansoura University Hospital, Egypt, 2021–2022

Studied variables	Study population (*n* = 133)
Age in years	
Mean (SD)	38.4 (9.5)
Min–max	17–64
Sex (no. (%))	
Male	25 (18.8)
Female	108 (81.2)
Education (no. (%))	
Basic	27 (20.3)
Technical school/institute	85 (63.9)
University	21 (15.8)
Job description (no. (%))	
Housekeepers	35 (26.3)
Nurses	98 (73.7)
Duration of employment in years	
Median (min–max)	16 (1–32)
Daily work hours	
Median (min–max)	12 (6–12)
History of HCV infection (no. (%))	4 (4.2)
History of HB vaccination (no. (%))	44 (60.3)
Previous waste management training (no. (%))	61 (45.9)

### Comparison of overall pre- and post-KAP scores

The overall knowledge, attitude, and practice scores among the studied healthcare workers were significantly higher after the intervention (*p* < 0.05) (Table 2).

**Table 2:** Comparison of overall knowledge, attitude and practice among study group before and after the intervention

Studied KAP	Pre Mean ± SD	Post Mean ± SD	Statistical significance (paired *t* test and probability “*p*”)
Total knowledge score	20.6 (3.08)	22.9 (2.4)	*P* < 0.001
Total attitude score	8.2 (1.5)	9.2 (0.9)	*P* < 0.001
Total practice score	20.3 (3.9)	23.1 (4.0)	*P* < 0.001

### Variation of pre- and post-knowledge scores according to different parameters

The pre- and post-knowledge scores were significantly different with respect to education, job description and duration of employment (*P* < 0.05). Moreover, knowledge scores were improved post-intervention compared to pre-intervention in all parameters (Table 3).

**Table 3:** Variation of pre- and post-knowledge scores according to different parameters

Variables	Studied knowledge		Statistical significance (paired *t* test and probability “*p*”)
	Pre Mean ± SD	Post Mean ± SD	
Sex:			
Male	19.6 ± 3.5	22.1 ± 2.6	*P* = 0.007
Female	20.8 ± 2.9	23.1 ± 2.4	*P* < 0.001
*Unpaired t test*	*P* = *0.06*	*P* = *0.07*	
Age in years:			
< 30	20.7 ± 2.9	23.1 ± 2.4	*P* = 0.007
≥ 30	20.3 ± 3.5	22.2 ± 2.4	*P* < 0.001
*Unpaired t test*	*P* = *0.5*	*P* = *0.07*	
Education:			
Basic	17.3 ± 3.4^AB^	21.07 ± 2.4 ^AB^	*P* < 0.001
Technical	21.3 ± 2.3^A^	23.3 ± 2.3 ^A^	*P* < 0.001
University	21.6 ± 2.4^B^	23.6 ± 1.6 ^B^	*P* < 0.001
*One way ANOVA test*	*P* < *0.001*	*P* < *0.001*	
Job description:			
Housekeepers	17.5 ± 3.2	21.02 ± 2.5	*P* < 0.001
Nurses	21.7 ± 2.2	23.6 ± 2.07	*P* < 0.001
*Unpaired t test*	*P* < *0.001*	*P* < *0.001*	
Duration of employment			
≤ 15 years	19.7 ± 3.4	22.3 ± 2.5	*P* < 0.001
> 15 years	21.4 ± 2.4	23.5 ± 2.3	*P* < 0.001
*Unpaired t test*	*P* = *0.001*	*P* = *0.004*	
Previous training			
Yes	20.7 ± 3.0	23.2 ± 2.3	*P* < 0.001
No	20.4 ± 3.1	22.6 ± 2.5	*P* < 0.001
*Unpaired t test*	*P* = *0.5*	*P* = *0.2*	

AB indicates the statically significant difference between groups (*p* < 0.05)

### Variation of pre- and post-attitude scores according to different parameters

The pre-attitude scores were significantly different with respect to education, job description and duration of employment with a statistically significant difference (*P* < 0.05). However, post-attitude scores were significantly different with respect to education and job description only (*P* < 0.05). Moreover, attitude scores were improved significantly post-intervention compared to pre-intervention in all parameters (*P* < 0.05) (Table 4).

**Table 4:** Variation of pre- and post-attitude scores according to different parameters

Variables	Studied attitude		Statistical significance (paired *t* test and probability “*p*”)
	Pre Mean ± SD	Post Mean ± SD	
Sex:			
Male	7.3 ± 2.05	8.9 ± 1.4	*P* = 0.002
Female	8.3 ± 1.3	9.3 ± 0.8	*P* < 0.001
Unpaired *t* test	*p* = 0.001	*p* = 0.09	
Age in years:			
< 30 (*n* = 30)	7.7 ± 2.3	9.3 ± 0.9	*p* = 0.001
≥ 30 (*n* = 103)	8.3 ± 1.3	9.2 ± 0.9	*p* < 0.001
*Unpaired t test*	*p* = *0.2*	*p* = *0.6*	
Education:			
Basic (*n* = 27)	6.5 ± 1.7 ^AB^	8.5 ± 1.3 ^AB^	*p* < 0.001
Technical (*n* = 85)	8.6 ± 1.2^A^	9.4 ± 0.7 ^A^	*p* < 0.001
University (*n* = 21)	8.4 ± 1.3^B^	9.4 ± 0.7 ^B^	*p* < 0.001
*One-way ANOVA test*	*p* < *0.001*	*p* < *0.001*	
Job description:			
Housekeepers (*n* = 35)	6.8 ± 1.6	8.6 ± 1.2	*p* < 0.001
Nurses (*n* = 98)	8.6 ± 1.2	9.4 ± 0.7	*p* < 0.001
*Unpaired t test*	*p* < *0.001*	*p* < *0.001*	
Duration of employment			
≤ 15 years	7.8 ± 1.8	9.1 ± 0.9	*p* < 0.001
> 15 years	8.5 ± 1.0	9.3 ± 0.9	*p* < 0.001
*Unpaired t test*	*0.006*	*0.2*	
Previous training			
Yes	8.1 (1.5)	9.3 (0.7)	*P* < 0.001
No	8.2(1.6)	9.2(1.1)	
*Unpaired t test*	*p* = *0.6*	*p* = *0.6*	

AB indicates the statically significant difference between groups (*p* < 0.05)

### Variation of pre- and post-practice scores according to different parameters

The total pre-practice scores were significantly different with respect to education and job description (*P* < 0.05). However, the post-practice scores were significantly different with respect to sex, age, education, and job description (*P* < 0.05). Moreover, practice scores were improved significantly post-intervention compared to pre-intervention in all parameters (*P* < 0.05) (Table 5).

**Table 5:** Variation of pre- and post-practice scores according to different parameters

Variables	Studied practice		Statistical significance (paired *t* test and probability “*p*”)
	Pre Mean ± SD	Post Mean ± SD	
Sex:			
Male	19.6 ± 4.2	20.7 ± 4.7	*P* = 0.2
Female	20.8 ± 3.8	23.7 ± 3.7	*P* < 0.001
*Unpaired t test*	*P* = *0.3*	*P* = *0.001*	
Age in years:			
< 30	19.5 ± 3.8	21.8 ± 4.8	*P* = 0.005
≥ 30	20.5 ± 3.9	23.5 ± 3.7	*P* < 0.001
*Unpaired t test*	*P* = *0.1*	*P* = *0.03*	
Education:			
Basic	18.2 ± 5.6^A^	20.6 ± 4.2^AB^	*P* = 0.01
Technical	21.02 ± 3.2^A^	23.7 ± 3.8^A^	*P* < 0.001
University	20.1 ± 3.3	24.1 ± 3.4^B^	*P* < 0.001
*One-way ANOVA test*	*P* = *0.006*	*P* = *0.001*	
Job description:			
Housekeepers	18.3 ± 5.2	20.7 ± 4.1	*P* = 0.004
Nurses	21.03 ± 3.1	23.9 ± 3.7	*P* < 0.001
*Unpaired t test*	*P* < *0.001*	*P* < *0.001*	
Duration of employment			
≤ 15 years	19.9 ± 4.2	22.5 ± 4.1	*P* < 0.001
> 15 years	20.6 ± 3.6	23.7 ± 3.9	*P* < 0.001
*Unpaired t test*	*P* = *0.3*	*P* = *0.1*	
Previous training			
Yes	20.9 ± 4.1	23.4 ± 3.7	*P* < 0.001
No	19.8 ± 3.7	22.9 ± 4.3	*P* < 0.001
*Unpaired t test*	*P* = *0.1*	*P* = *0.5*	

*AB* indicates the statically significant difference between groups (*p* < 0.05)

## Discussion

This quasi-experimental study found significant improvement in the KAP scores post compared to pre-intervention. The post-knowledge and attitude scores were significantly better in nurses and participants with a higher education. The post-practice scores were significantly better for females, participants with an age ≥ 30 years, higher education, and nursing jobs.

### Knowledge scores

The current study results revealed that knowledge scores were significantly higher after the intervention. These results agree with El-Naggar et al. (2017), who found a significant difference between pre- and post-intervention scores among physicians, nurses, technician, and auxiliary workers at Zagazig University hospitals [8].

#### Variation of knowledge according to different factors

The current study results revealed that both pre- and post-knowledge scores were significantly higher among nurses compared to housekeepers. Also, knowledge scores were significantly higher among workers attaining technical and university education than basic education.

Similarly, Soyam et al. (2017) in India reported that the overall knowledge of HCWs was high but the nursing staff was excellent. This could be explained by the fact that most of the study subjects received superior training on biomedical waste management (BMWM) frequently and approximately half of the HCWs had received training within 1 year. Also, in the same study, workers with a diploma in general nursing midwifery (GNM) had a higher level of knowledge on BMWM than other professionally qualified workers. Technical staff had significantly lower knowledge scores on BMWM than all nursing staff [6].

In addition, previous studies in India reported that the difference in the knowledge of nursing staff regarding the color coding system of bags for bio-medical waste was statistically significant [6, 9]. Nursing staff know much better in which bag the infected waste should be disposed of than other workers. This was attributed to the fact that nurses were more involved in applied work and tasks given by higher authorities [10].

The results of the present study are different from those revealed by another study from India, which found that the knowledge of the existence of biomedical waste management rules was better among doctors than nurses or paramedical staff, but that knowledge of the practical aspects of biomedical waste management was better among nurses and paramedical staff [11].

Hakim et al. (2014) assessed the KAP of health-care providers towards waste management at Ain Shams University Hospitals, Egypt and reported that knowledge about the presence of department plans and a hospital system for waste handling was significantly better among housekeeping staff than among nurses or physicians. On the other hand, the housekeeping staff in their study were less knowledgeable about specific details of management [5].

With regards to the association between the duration of employment and overall knowledge, the current study found higher levels of knowledge score among senior workers. Similarly, previous studies have reported that the years of experience in the hospital is significantly correlated with the level of knowledge [6, 12].

In addition, a study carried out in Iran, found that knowledge was highest in the 30 to 40 years’ age group and lowest in the group aged more than 50 years old [13]. Moreover, another recent study reported that the age and work experience of the study participants were significantly associated with knowledge regarding biomedical waste management (BMWM) [14].

However, another study found that younger workers (26–30 years) had higher knowledge of BMWM [6]. These conflicting findings may be attributed to the adequacy and frequency of training sessions conducted in the respective study settings [14]. In general, most of the literature agrees that knowledge regarding biomedical waste management was better in older and more experienced staff.

#### Variation of attitude according to education and job description

In the present study the post-attitude scores were significantly higher in workers with technical and higher education compared to basic education. Similarly, a previous study revealed that diploma holders had better attitude than educated workers with lower degrees [15]. Also, Soyam et al. (2017) in India found that senior secondary level educated respondents were more compliant with BMWM [6].

In agreement with the present study results, which found that nurses have a significantly higher knowledge score than housekeepers, a study in three hospitals at Menoufia Governorate, Egypt, where the majority of nurses (89.4%) in the study settings had high level of knowledge and high performance about hospital waste management [16].

#### Variation of practice according to different factors

The present study revealed that practice scores were significantly higher post-intervention compared to pre-intervention and the variation of both the pre- and post-practice scores were significantly noticed in nurses more than housekeepers.

Improvement of practices scores obtained in the current study are consistent with results of Pratinidhi et al. (2014) who reported that in pre-training observation there were 83.9% of biomedical waste handlers in the poor practice category which decreased to 2.1% post-training [17].

In addition, Hosny et al. (2018) showed that the 80.0% of the pre-training poor practice score changed to 0.8% post-training and that the 1.1% in the good practice category increased to 92.1% post-training [18].

Moreover, a study in India found that the practice scores of nurses were significantly higher than those of physicians (84.8% versus 67.3% had overall satisfactory practice). The authors explained this difference with the lack of training, as fewer physicians had received training on proper waste management during the course of their study [6].

Also, Gupta et al. (2015) revealed that the practice score was poor in 62% of sanitary workers and was average in 38% of them. After application of the training program, there was a significant decrease in the number of subjects who had poor practice scores (from 80 to 0.8%). These findings highlighted that the educational intervention was very effective and may be attributed to the clarity and direct applicability of the practical skills offered in such programs [19].

In the present study there was no significant association between previous waste management training and a change in KAP scores after intervention. These results came in agreement with previous research which confirmed that this does not reduce the importance of training courses and orientation programs on awareness about waste management, it does however raise an important question regarding the ability to improve practice in healthcare workers [5].

Another cross-sectional study in Egypt showed that, although attendance of training programs on waste management was the only statistically significant independent predictor of health-care staff’s knowledge, no such correlation was found with actual practices [20]. Another aspect that could affect the impact of training programs in the present study are temporary jobs, lower education levels for housekeepers and high turnover of nurses.

Researchers have suggested that most training courses and orientation programs emphasize theoretical aspects with numerous lectures but minimal hands-on training. Moreover, training programs should take into consideration the educational level of housekeepers, since a significant proportion is illiterate in developing countries [20, 21]. Finally, intervention studies play an important role in reflecting the effect of waste management programs on the KAP of workers. Most of them can improve the KAP of healthcare workers taking into account national guidelines and challenges.

### Strengths and limitations of the study

This study is an intervention to test the effect of an educational program on the KAP of HCWs, explore possible factors that may affect their KAP and recommend the measures required for best practice in waste management. However, the study did not include physicians and was a single center study. The Cronbach’s alpha for the tool is moderate. Rapid turnover & lower educational levels of housekeepers are challenging for hospital administrations with regard to the application and evaluation of the immediate outcomes from training programs.

## Conclusion

There was a significant improvement in the KAP scores post-intervention. Post-knowledge scores were significantly better in nurses, higher education participants, and participants with a longer duration of employment. Post-attitude score was significantly better in higher education participants and nurses. Post-practice score was significantly better in females, participants aged ≥ 30 years, participants with higher education, and nurses.

Continuous yearly educational programs are recommended by occupational health and safety teams for the retention of knowledge. The combination of training and supervision are crucial for the success of waste management programs to correct malpractice. Higher education levels are required for housekeepers to be capable of gaining better knowledge, follow rules and be ready for any challenges in the future.

## Data Availability

Data is available in the Harvard Dataverse Repository: Effect of waste management intervention program on knowledge, attitude, and practice (KAP) of nurses and housekeepers: a quasi-experimental study in Mansoura Emergency Hospital, Egypt. It is accessible through the following link: https://dataverse.harvard.edu/dataset.xhtml?persistentId=doi:10.7910/DVN/TN3UNW—Harvard Dataverse (Khashaba, 2023).

## Abbreviation

BMWM: Biomedical waste management
HWM: Healthcare waste management
KAP: Knowledge–attitude–practice
GNM: General nursing midwifery
